# Medical education during the COVID-19 pandemic: lessons for the orthopedic departments

**DOI:** 10.1186/s12909-023-04388-w

**Published:** 2023-06-13

**Authors:** Zahra Vahdati, Hossein Nematian, Amir Reza Farhoud, Mohammad Naghi Tahmasebi, Shahram Rahimi-Dehgolan, Seyed Mohammad Javad Mortazavi, Reza Shahryar Kamrani, Leila Oryadi Zanjani, Mohammad Reza Golbakhsh, Roya Nasl Seraj, Mohammad Hossein Nabian

**Affiliations:** 1 School of Medicine, Tehran University of Medical Sciences, Tehran, Iran; 2 Center of Orthopedic Trans-Disciplinary Applied Research (COTAR), School of Medicine, Tehran University of Medical Sciences, Tehran, Iran

**Keywords:** COVID-19, Distance education, Education, Medical education

## Abstract

**Background:**

After the Coronavirus pandemic, many educational routines were stopped for the safety of medical staff. To achieve educational goals, we have implemented new policies in our hospitals. In this study, we aimed to evaluate the effect of such strategies.

**Method:**

This survey-based study uses questionnaires to assess newly implemented educational strategies. We surveyed 107 medical staff of the orthopedic department of Tehran University of Medical Sciences, including faculty members, residents, and students. The survey contained three series of questionnaires for these groups.

**Results:**

The maximum satisfaction for all three groups was observed in the platform and facilities for using e-classes, and the cost- and time-saving capabilities (Respectively, faculty members (FM): 81.8%, residents (R): 95.2%, students/interns (S/I): 87.0%; FM: 90.9%, R: 88.1%, S/I: 81.5%). The new policies have been shown to reduce the stress level of most trainees, increase the quality of knowledge-based education, increase the opportunity for reexamining educational content, expand discussion and research opportunities, and improve work conditions. There was a broad acceptance of the virtual journal clubs and morning reports. However, there were discrepancies between residents and faculty members on issues such as the evaluation of trainees, the new educational curriculum, and flexible shift schedules. Our strategies failed to improve skill-based education and patient treatment status. Most participants indicated that e-learning should be used with face-to-face training post-pandemic (FM: 81.8%, R: 83.3%, S/I: 75.9%).

**Conclusion:**

Our efforts to optimize the educational system during this crisis have generally improved trainees’ work conditions and educational experience. Most participants believed that e-learning and virtual methods should be used alongside traditional training as a complementary component after the pandemic.

**Supplementary Information:**

The online version contains supplementary material available at 10.1186/s12909-023-04388-w.

## Background

The coronavirus disease 2019 (COVID-19) has imposed a massive burden worldwide despite appearing quickly. As the leading clinical education providers, hospitals became centers focusing on COVID-19, forcing medical education to shut down in-person classes and change many educational routines [1–5].

At Tehran University of Medical Sciences, three general hospitals (Shariati Hospital, Imam Khomeini Hospital, and Sina Hospital) were the leading referral centers for trauma patients. However, they admitted many COVID-19 patients during the crisis. As a result, many educational routines traditionally held at these hospitals and university hospitals for many years were stopped for the safety of medical staff. As a result of the COVID-19 pandemic, referral and educational hospitals were among the leading centers to deal with these conditions at the beginning of the outbreak. Some of these programs in the orthopedic department are daily bedside rounds, one-hour morning reports five days a week, focused group discussions, and the 40-year tradition of weekly grand rounds, research proposals, and defense of residents’ or fellow theses [6].

The Internet infrastructure has brought valuable benefits to the modern educational system. Tele-education is now available worldwide owing to the high-speed internet and smart devices. Many innovative and user-friendly applications are available for electronic devices that have made it possible to work or educate from home or anywhere in the world. In addition, they have created the opportunity for a group or personal learning in offline and online environments [7–12].

To achieve educational goals during the crisis, it was essential to have new plans and approaches based on existing potentials. Considering that the knowledge of orthopedics has many practical procedures, virtual education in this field requires detailed investigations. Here, we describe new policies implemented in the orthopedic department of Tehran University of Medical Sciences during the COVID-19 crisis. In addition, we assessed trainers’ and trainees’ opinions about the effects of new strategies on their knowledge and skills, especially the use of e-learning and virtual solutions.

## Methods

### Implemented strategies

Since the outbreak of COVID-19 in February 2020 in Tehran, various e-learning methods have been established to reduce the presence of residents and students in hospitals, primarily until June 2021. The following strategies were implemented [Table 1]:

**Table 1:** Summary of the implemented strategies during the COVID-19 crisis

Domain	Strategy
Limiting person-to-person contact	Exemption of medical students and interns from presence in the hospital
	Attribution of each patient to one physician (faculty member, fellow, and resident)
	Suspension of the hierarchical system among residents
	Flexibility of night-shift schedule
	Reduction of the hand-written documentation
	Preparation of educational videos about updated personal protection principles and detailed instructions on the equipment
Educational policies	Coordination of daily shifts via messaging applications and chat group
	Educating medical students and interns in a separate chat group
	Running daily morning reports via a chat group
	Holding a one-hour video conference with an interactive approach via Skype
	Preparation of education packages for medical students and interns followed by a virtual class with group discussion
	Reviewing book chapters during one-hour online sessions three times per week
	Holding online journal club sessions one hour per week
Trainees’ assessment & graduation	Assessing trainees at all levels with online exams
	Virtual dissertation defense for interns and residents
Research	Online video-conferencing of the education and research council for brainstorming and decision making

#### Limiting person-to-person contact

- Medical students and interns were exempted from their presence in the hospital, including orthopedic and emergency wards, physical classes, and operating rooms. To prevent the learning gap, the full potential of e-learning was utilized. Interns who volunteered to serve on the emergency and orthopedic wards were registered as backups in critical situations.
- To decrease unnecessary exposure, the trend was to assign each patient to one physician (including faculty member, fellow, and resident) based on the physician’s experience in a specific field. The on-call faculty members were mainly responsible for clinical decisions and patient monitoring.
- The hierarchical system among medical residents was suspended, and all grades could contact the responsible faculty member directly.
- The night-shift schedule was flexible so that after any possible exposure, home isolation of personnel for 14 days was feasible.
- On the orthopedic and emergency wards, hand-written documentation was reduced as much as possible. In addition, all records and orders were stored in digital formats such as voice and photographs to reduce contact with folders and papers.
- Updated personal protection principles and detailed instructions on the equipment were provided in educational videos.

#### Educational policies

- Daily communication among physicians of each shift was done through messaging applications (e.g., WhatsApp, Skype, Sky Room) via group chats, while other members could watch the conversations. Therefore, residents could learn and discuss issues like diagnosis, decision-making, surgical techniques, intraoperative pictures, possible complications, and outcomes.
- Fellows and residents were responsible for educating and examining the medical interns and students in separate groups and reporting the progress to a determined attending surgeon.
- Daily morning reports were conducted, and the material of morning reports was discussed by text and voice messages, as well as required videos and pictures uploaded to a chat group before the session.
- At a determined time in the morning, a one-hour video conference was held via Skype five days a week. Other team members had the opportunity to observe and discuss all management plans.
- The education curriculum topics for medical students and interns were presented in educational packages. Each package contained a PowerPoint presentation file, a voice or video of the lecturer, and a self-assessment quiz. In addition, an interactive virtual class with a group discussion was held after the presentation.
- A one-hour online book review session was held under the supervision of an attending surgeon every other day, moderated by a fellow or resident.
- Journal clubs continued with the old routine of one hour per week via online methods.

New educational interventions are also described using the GREET statement in Table 2 [13, 14].

**Table 2:** Summary of Educational Interventions based on GREET Statement

Brief Name	Intervention	We conducted a survey-based study to evaluate new educational strategies based on virtual learning that we applied during the COVID-19 pandemic.
Why	Learning Objectives	The purpose of using these educational strategies based on virtual education during the COVID-19 pandemic was to continue education and research with high efficiency, despite maintaining the health and safety of medical staff.
	Theory	Virtual education strategies were implemented by faculty members in the orthopedics department of our university in May 2021, almost two months after the COVID-19 pandemic. These strategies were implemented to reduce person-to-person contacts and continue education and research processes. Moreover, feedback on these methods was collected from the medical staff. Therefore, this survey aimed to ensure that educational audiences find these methods helpful.
What	Materials	Virtual training content was provided in the form of voice, picture, and texts in messaging applications such as WhatsApp or educational packages, including a PowerPoint file and a voice or video presentation and self-assessment quiz on the site, classes or group discussions, morning report sessions or journal club sessions in Video communication applications such as Skype or Sky room.
	Educational Strategies	Implemented educational strategies were explained in the form of 4 parts limiting person-to-person contact, educational policies, Trainees’ assessment and graduation, and research in [Table 1].
	Incentives	Participants were not paid, and there were otherwise no financial incentives.
Who	Instructors	The educational strategies were designed and implemented with the opinion and consultation of orthopedic faculty members at all levels (assistant professor, associate professor, and professor). The audience of these educational strategies was the residents and students of the orthopedics department.
How	Delivery	Virtual training materials were provided through online platforms, messaging and video communication applications, and offline content. Meetings and classes were held via video conference. The exams were held through an external internet connection.
Where	Environment	The faculty members prepared the educational content mainly at home or in their office in the hospital, and the medical students listened to the virtual education content at home. The residents also listened to these contents at home or in the orthopedic department with the changes applied in their work shifts. In addition, medical staff participated in online meetings, mostly at home or sometimes in their office at the hospital.
When, How much	Schedule and Time	Virtual education strategies were implemented in May 2021, almost two months after the COVID-19 pandemic. The education curriculum topics for medical students were presented in educational packages; each package was presented over a 2-hour offline file. In addition, an interactive virtual class with a group discussion was held after the presentation in one hour. Daily morning reports were conducted as a one-hour video conference five days a week. A one-hour online book review session was held under the supervision of an attending surgeon every other day, moderated by a fellow or resident. Journal clubs were conducted one hour per week via online methods.
Planned Changes	Specific Adaptation	Since the outbreak of COVID-19 in February 2020 in Tehran, various e-learning methods have been established to reduce the presence of residents and students in hospitals; in order to adapt the educational conditions to this situation, it was necessary to provide internet infrastructure and virtual means of communication in order to be able to present the prepared educational materials to the learners and encourage them to use virtual education and schedule.
Unplanned Changes	Modification	There were no unplanned changes.
How Well	Attendance	All the students had to confirm the study of educational packages on the site and in their accounts. The orthopedics department’s educational expertise also recorded learners’ attendance in virtual classes and meetings. Moreover, exams and quizzes were also held online to evaluate the trainees.

#### Trainees’ assessment and graduation

A previously designed online platform was used to conduct all planned exams. In addition, it was modified to be accessible via an external internet connection, making it possible to take exams from home.

- The dissertation defense is a mandatory prerequisite for introducing residents to the final licensing exam and board certification. As the endpoint of the COVID-19 pandemic is not predictable, we decided to attend defense sessions virtually. Interestingly, compared to in-person sessions, the pressure was much lower during an online defense, and we could assess many details more precisely.

#### Research

- The education and research council of the orthopedic department was able to continue its work through videoconferencing. During these sessions, the members were eager to share their ideas about the various aspects of crisis management. Sometimes, a difficult situation might speed up innovation and force an old system to give up inherited habits.

### Study design and participants

This survey-based study was conducted at the Tehran University of Medical Science, Tehran, Iran, from May 10, 2021, to July 31, 2021. This survey was done 6 weeks after implementing new educational interventions according to the conditions of the COVID-19 pandemic. During this two-month study, one hundred and seven medical staff of the orthopedic department of Tehran University of Medical Sciences who were present in our educational hospitals during the study and changes in educational strategies were conveniently invited to participate. Due to the lack of data on this issue at the beginning of the COVID-19 crisis and the small number of participants, we surveyed author-made questionnaires. The staff was divided into three groups: (a) faculty members (n = 11), (b) residents (n = 42), (c) interns/students (n = 54; 11 interns, 43 students). The survey contained questionnaires for these three groups with 22, 22, and 15 questions, respectively [Appendix 1].

#### Study population

The study population was the medical staff of the orthopedic department of Tehran University of Medical Sciences in all three referral and educational hospitals (Imam Khomeini Hospital Complex (IKHC), Sina Hospital, and Shariati Hospital). The eligibility criteria for participants were:

1. Faculty members of the Department of Orthopedics of Tehran University of Medical Sciences at all levels (assistant professor, associate professor, and professor).
2. Orthopedic residents of Tehran University of Medical Sciences at all stages from the first year to the fourth year.
3. 6th and 7th-year medical students in the orthopedics department (as orthopedic interns).
4. 4th and 5th-year medical students in the orthopedics department (as orthopedic students).

All eligible people; one hundred and fifty-one medical staff of the orthopedic department of Tehran University of Medical Sciences who were present in our educational hospitals during the study and changes in educational strategies were conveniently invited to participate in this survey to fill out the questionnaire. 11 out of 27 faculty members (40.74% response rate) and 42 out of 56 orthopedic residents (75% response rate) and 54 out of 68 medical students (79.41% response rate) participated in this survey. A total of one hundred and seven medical staff participated in this survey.

### Study questionnaire

The questionnaire was developed through a Delphi technique, which seeks experts’ opinions to assess the extent of agreement and resolve disagreement on challenging issues.

The Delphi technique process comprised three rounds. First, participants were asked to rank 24 statements independently, using a 9-point Likert scale in round 1. The free-text response was also available within each of the seven survey domains to provide the opportunity to elaborate or explain responses.

In round 2, each participant received an individualized survey that comprised 27 statements. Round 2 survey included 24 statements from round 1, which were presented alongside participants’ responses and the group’s collective response from round 1. Participants were asked to reconsider their responses in light of the group’s responses. The second-round survey also included three new statements derived from the free-text responses from round 1.

In Round 3, each participant received an individualized survey comprising 27 statements from Round 2, which were presented alongside the participants’ responses and the group’s response from Round 2. Participants were asked to reconsider their responses in light of the group’s responses a final time. There were no new statements in round 3.

The consensus was achieved on all except two statements with a response split, meaning consensus would be unlikely after the third survey round. All surveys were administered using Google Forms, and survey links were emailed.

### Survey development

Statements for the survey were developed from intelligence from the “School of Medicine - Tehran University of Medical Science Network” (which includes students, residents, and faculty members in medical sciences), a review of the literature, and the study team’s expertise. Statement development capitalized on an existing survey as part of “School of Medicine - Tehran University of Medical Science Network” activities. Members’ responses: ‘What are your concerns about the new educational conditions after the COVID-19 Pandemic?‘ Moreover, ‘What are the main challenges related to the new educational conditions after the COVID-19 Pandemic?‘ Five authors (M.N, Z.V, H.N, S.R, and L.O.) analyzed the responses independently to propose statements. These statements were refined in light of the knowledge of the research team and literature review findings.

A total of 27 statements were included across seven domains: [1] facilities and platforms for participating in e-courses, [2] quality of education in orthopedic knowledge and skills, [3] possibility of discussions, interactions, evaluations, and feedback, [4] educational and research activities outside the program, [5] the effect of shift schedules and training curriculum changes, [6] time and cost consumption, and [7] stress level. The survey statements were constructed to highlight the key challenges relating to each domain for each group and to achieve effective approaches to address them.

### Expert panel recruitment

A non-probability purposive sample of twelve experts from attending orthopedic professors and educational experts of the Tehran University of Medical Sciences were invited via email. Participants must respond across all three rounds to complete the Delphi process. A dropout rate of 20% was expected over the three rounds, in accordance with previous Delphi studies [15, 16].

### Data collection

Final questionnaires were presented to all 107 participants via email, and the data was collected in Excel format.

### Ethics

#### Ethical approval

for this study was granted by the Institutional Research Ethics Committee School of Medicine- Tehran University of Medical Sciences, and informed consent was obtained from all the students who participated in the study at the beginning of the process as part of the online survey.

### Data analysis

The consensus was achieved when > 70% of participants agreed/strongly agreed or disagreed/strongly disagreed with a statement in round 3. This level of agreement has been considered appropriate in previous Delphi studies [16, 17]. Descriptive statistics were used to describe group responses to each statement in all three groups. The data were analyzed using SPSS version 26.0 for Windows (SPSS, Inc., Chicago, IL).

### Outcomes

This research aims to understand the impact of virtual orthopedic training on medical staff and how this form of training can be effectively implemented after the pandemic. The findings of this research could help inform how best to provide medical staff with the necessary training in a time- and cost-effective manner. This research will also help to identify potential challenges and obstacles related to virtual orthopedic training that may need to be addressed in order to ensure successful implementation. It will also provide insights into how virtual orthopedic training can be improved in the future.

The primary outcome was to evaluate the impact of the COVID-19 pandemic on educational schedules, educational quality, and the level of satisfaction of medical staff with their educational situation. As a secondary outcome, virtual orthopedic training was examined for its effect on time and cost consumption and stress levels among medical staff.

## Results

To design questionnaires using the opinions of faculty members and experts and to use the Delphi method for this purpose, of the 12 experts invited to participate in this Delphi study, 9 participants completed Round 1 (75% response rate), 8 of 9 completed Round 2 (88.8% response rate) and 7 of 8 completed Round 3 (87.5% response rate).

The final questionnaires [Appendix 1] included eight joint statements between all three participating groups, nine joint statements between residents (R) and faculty members (F.M.), four joint statements between students/interns (S/I) and faculty members, and three joint statements between students/interns and residents. In addition, there was one unique statement for faculty members and two unique statements for residents in the final questionnaires. The final questionnaires and the answers’ results are diagrams in Appendix 2.

**Fig. 1:**
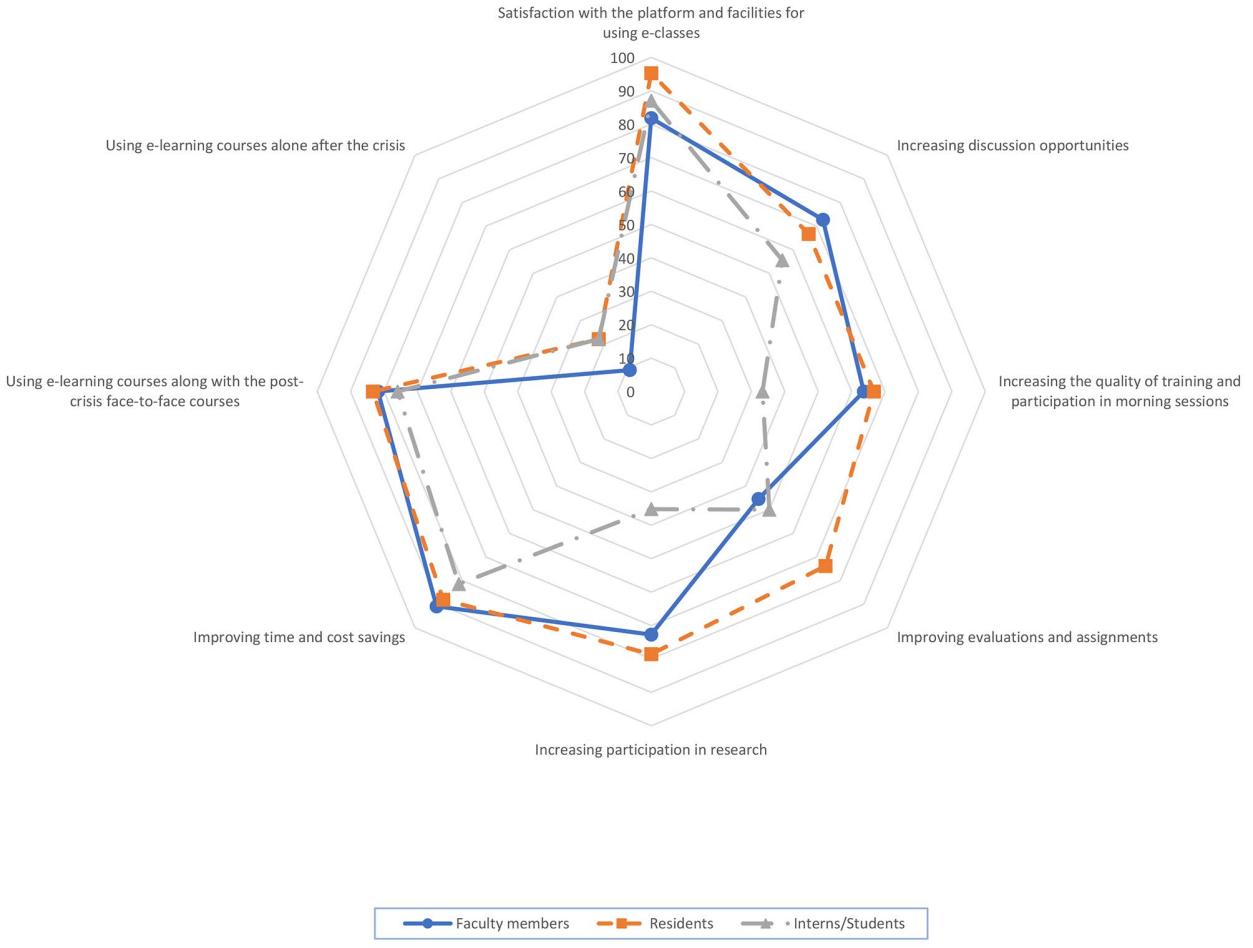
Common statements between all 3 participating groups, (**a**) faculty members (n = 11), (**b**) residents (n = 42), (**c**) students/interns (n = 54)

Three statements on “satisfaction with e-learning infrastructure,“ “cost-time savings,“ and “use of e-learning along with face-to-face training in the post-pandemic period” among the joint statements among all participants had the highest agreement in all three groups (respectively: FM: 81.8%, R: 95.2%, S/I: 87.0%; FM: 90.9%, R: 88.1%, S/I: 81.5%; FM: 81.8%, R: 83.3%, S/I: 75.9%) [Fig. 1].

There was agreement among participants that e-learning could increase discussion opportunities (FM: 72.7%, R: 66.7%, S/I: 55.5%). However, approximately half of the faculty members and students stated that this method had not improved the trainees’ evaluation, while residents favored it (FM: 45.5%, R: 73.8%, S/I: 50.0%). Also, the quality of training and participation in morning reports has slightly increased while a relatively small number of students and interns have expressed satisfaction with this issue. (FM: 63.6%, R: 66.7%, S/I: 33.3%). Finally, research activities have been improved with new strategies, as stated by faculty members and residents. However, only 35.2% of interns and students agreed that this had increased research participation (FM: 72.7%, R: 78.6%, S/I: 35.2%) [Fig. 1].

The idea of using e-learning courses along with in-person courses after the pandemic was highly favorable among all groups. On the contrary, exclusive use of e-learning courses without in-person courses was unacceptable (FM: 9.1%, R: 50%, S/I: 22.2%) [Fig. 1].

**Fig. 2:**
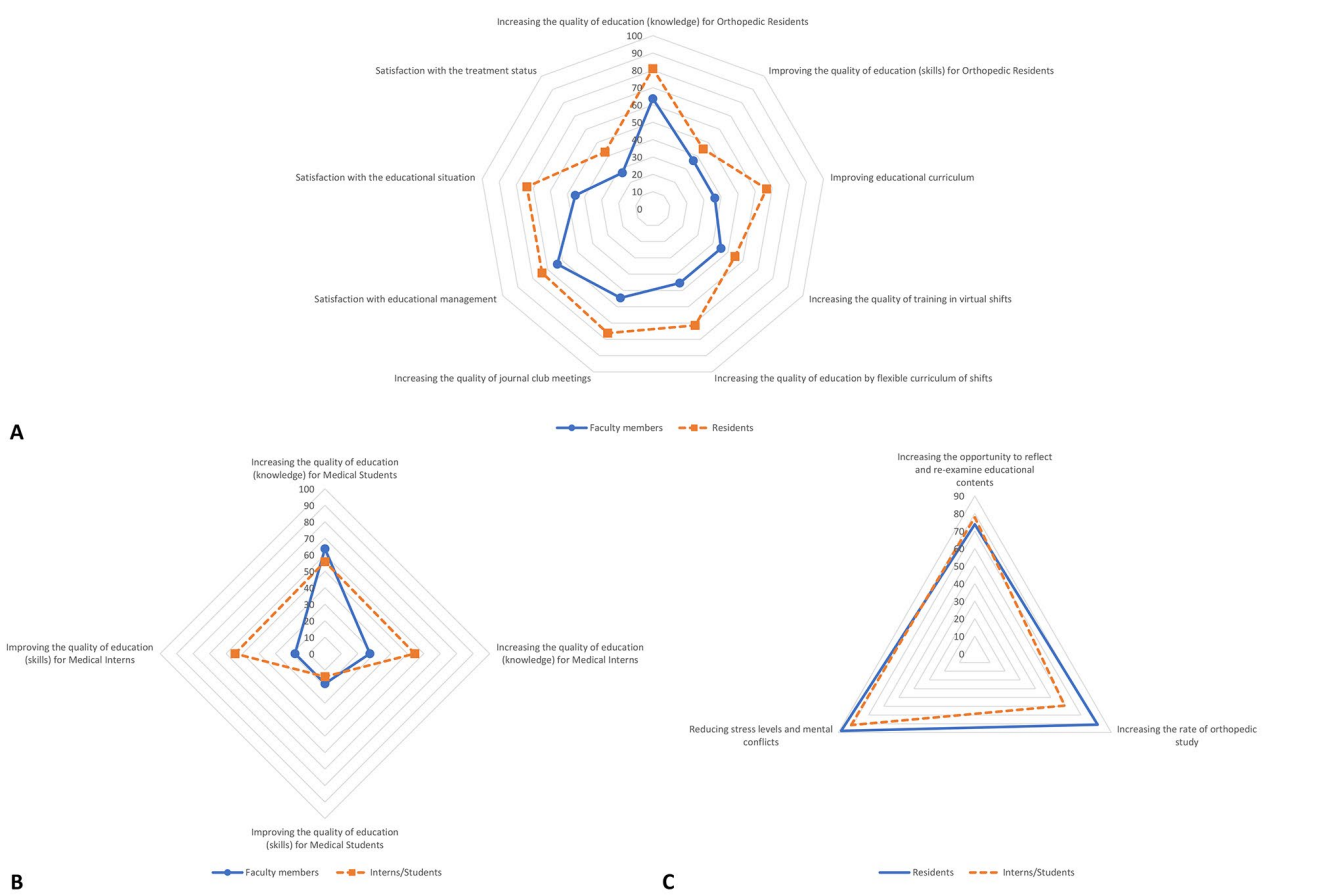
**A.** Common statements between residents (R) and faculty members (FM), **B.** Common statements between students/interns (S/I) and faculty members, **C.** Common statements between students/interns and residents. (**a**) faculty members (n = 11), (**b**) residents (n = 42), (**c**) students/interns (n = 54)

Regarding the impact of e-learning on the quality of knowledge-based education, there was relative agreement among students, interns, and residents to use this method (S: 55.8%, I: 54.5%, R: 80.9%). Furthermore, faculty members’ views on increasing the quality of knowledge-based education among students and residents were similar to those of the participants (63.6% and 63.6%). However, faculty members disagreed with the positive impact of e-learning on knowledge-based education for interns (27.3%). In addition, there was general dissatisfaction among trainers and trainees regarding the quality of skill-based education with this method. Surprisingly, however, the interns believed in the positive effect of using this educational method on the quality of skill-based education (54.5%) [Fig. 2A and B].

The results of the joint statements between faculty members and residents show more remarkable relative agreement among residents than among faculty members. Most participants believed that the quality of journal clubs has increased (FM: 54.5%, R: 76.2%). A majority of residents stated that two items of work conditions, including “quality of training in virtual shifts” and “quality of education by a flexible curriculum,“ have been improved with new strategies (respectively 54.8% and 71.4%). There was no agreement between faculty members and residents regarding improving the educational curriculum with this method (FM: 36.4%, b: 66.7%). The highest rate of satisfaction among residents was related to educational management (73.8%) and educational situation (73.8%), while the faculty members were delighted with research management (72.7%). The lowest satisfaction rate was detected in the patient’s treatment (FM: 27.3%, R: 42.9%) [Fig. 2A]. About 61.9% of residents believed that communication with the faculty members under these circumstances had increased. They also believed that reducing attendance at the ward led to increased learning of orthopedic contents (66.7%).

Some common questions were also asked of residents and students/interns. More than 70% of trainees believed that e-learning had increased the opportunity for reexamining educational content (R: 73.8%, S/I: 77.8%). They have also stated that the new policies have reduced the stress level of most trainees (R: 88.1%, S/I: 81.5%). About 59.3% of students/interns and 81.0% of residents have stated that their orthopedics study has increased during this period [Fig. 2 C].

## Discussion

This study aimed to evaluate the virtual educational methods used during the COVID-19 pandemic in the orthopedic department. According to our results, the participants were generally satisfied with the platform and electronic equipment for e-learning. These methods showed great potential for saving time and cost and reducing staff stress levels. Other studies by Kogan et al. and Wong et al. have shown that stress and burnout are higher in critical situations like a pandemic. This is due to the lack of personal protective equipment, training to deal with the new problem, and the risk of infecting loved ones [18, 19]. Gallagher et al. believe that medical students are also dramatically affected by this crisis, and even the feeling of anxiety may dominate the commitment to serve the sick.

Moreover, they are worried about their education, and in many cases, the high risk of infection outweighs the educational benefits of direct participation by students [20–22]. Any facilitation strategy should be utilized to decrease stress levels and burnout. Virtual sessions and e-learning may decrease the tension that face-to-face classes or dissertation defense sessions may impose on the medical staff.

Using e-learning methods has satisfied many residents and students who found new opportunities to study and review the educational content and engage more with their research activities. The new electronic format of the journal club during this pandemic has improved the research skills of our orthopedic residents and their critical appraisal capability for scientific research. The simplicity and availability of the electronic journal club can potentiate implementing this method in any educational center [23]. Although the faculty members believed that the quality of journal clubs and morning reports had mainly stayed the same compared to the past, the residents were delighted with the quality of virtual sessions.

Despite the agreement on the benefits of this educational method among all three groups, there were some differences between their viewpoints. For instance, most residents and faculty members reported increased opportunities for discussion through virtual platforms. At the same time, the students thought otherwise, probably because they were primarily not deeply involved in the discussions. Unlike residents, faculty members were unsatisfied with the educational curriculum during the COVID-19 crisis. Faculty members and interns/students did not consider the new method of evaluation and assignment as a strength of this strategy. This was in contrast to most residents who believed it was beneficial. Kogan et al. and Lewis et al. reported successful assessments of medical students through oral exams, video-supervised sessions, and online tests [18, 24]. On the other hand, teaching clinical skills was greatly affected during the crisis because of the distancing protocols, and clinical skill assessment tests like Objective Structured Clinical Examinations (OSCEs) were canceled [25].

Although faculty members were reasonably satisfied with the quality of trainees’ knowledge, they were vehemently opposed to learning clinical and practical skills through e-learning. Their point of view was similar to students and residents who have expressed concern about the quality of clinical skill learning. Woollisroft believes that due to this pandemic, medical students are removed from direct patient contact and skill learning, a crucial and essential component of their education [26]. Other experts have expressed similar concerns and highlighted that reducing trainees’ physical presence can limit their overall experience and education [27–30]. The media have extensively covered the heavy impact of COVID-19 on the lives of healthcare providers such as general practitioners, nurses, physician assistants, and faculty members. However, only one group has been left behind. Medical students are rarely mentioned despite being significantly affected by this crisis. We cannot predict how long this pandemic will last, so neglecting students and undergraduates could have severe consequences. Thus, we should continue medical education and put more emphasis on all aspects of this issue [31, 32].

This semester, we have increased residents’ work and learning quality. This is done by establishing a flexible shift program and eliminating the hierarchy of residents with direct access to faculty members. Although the residents agreed with the new curriculum, less than half of the faculty members were satisfied. Other studies have reported that surgery trainees faced significant challenges during the COVID-19 crisis due to the significant reduction in elective surgeries. Elective surgeries are great learning opportunities for assistants since they are usually performed by residents with little supervision, as opposed to complex or emergency surgeries performed mainly by attending surgeons [33–35]. In addition, canceling conferences, congresses, and other face-to-face meetings has reduced opportunities for continuing medical education [36]. The discrepancy between attending physicians and residents can be explained by the fact that residents are more concerned with reducing workload, which becomes more applicable with diminished attendance and a flexible shift schedule.

On the other hand, attending surgeons are more focused on teaching practical skills that need the physical presence of the residents in the hospital [35, 37–39]. Therefore, while residents were delighted with educational management and educational conditions, faculty members were generally dissatisfied with these aspects of the modified teaching strategies. The dissatisfaction of both groups with the treatment of patients is a concerning point that needs further investigation in a future study.

Overall, Kevin et al. have stated that we must continue to develop technologies, such as virtual meeting platforms and distance learning, to open up the new world of orthopedic education. This is just like our new strategies [40]. Moreover, as Wright et al. have expressed, this crisis offers us opportunities to be flexible and innovative and to learn in ways that many of us have never learned before [41]. Bagherifard et al. found that e-learning reduces stress levels and saves time, much like our findings in this study. They have also stated that orthopedic doctors had the opportunity to tend to their everyday lives, which they usually did not have time for. In addition, they had the opportunity to participate in other aspects of education, such as in our survey and research projects [42]. However, in addition to these essential benefits, the COVID-19 crisis and e-learning affected all levels of education, such as residents, interns, and trainees. It reduced practical exposure, canceled exams, and adjusted the curriculum, just as the Rupen study in the U.K. points out [43]. Considering the advantages and disadvantages of this new paradigm during the COVID-19 crisis, most participants believed that it was beneficial to use e-learning along with the face-to-face teaching method after this crisis.

***Suggestions***:

- Using e-learning and virtual methods alongside traditional training as a complementary component after the COVID-19 crisis.
- Using the platform of online classes and other virtual programs for programs that can be held online, such as morning reporting sessions and the club magazine.
- Holding in-person programs that require the direct presence of staff and will have a poor quality of training in virtual sessions, such as rounds and practical training classes.

***Limitations***:

- Limitations of this study include inadequate access to participants due to pandemic conditions and inadequate cooperation from some participants. In addition, our results might not be extrapolated due to the small sample size.

## Conclusion

Following the COVID-19 pandemic, our efforts to optimize the educational system during this crisis have reduced the stress level of the staff, helped with cost-saving and time management, increased study opportunities and research activities, and established virtual journal clubs and virtual morning reports. However, it had disadvantages, such as reduced clinical exposure, skill learning, and shortcomings in assessing practical skills. Therefore, most participants believed that e-learning and virtual methods should be used alongside traditional training as a complementary component after the COVID-19 crisis.

## Electronic supplementary material

Below is the link to the electronic supplementary material.

Supplementary Material 1

Supplementary Material 2

## Data Availability

The datasets generated and/or analyzed during the current study are available from MHN (dr.nabian@gmail.com) upon reasonable request.
