# Supplementary material for: Medical education during the COVID-19 pandemic: lessons for the orthopedic departments
Source: BMC Med Educ. 2023 Jun 13;23:436. doi: 10.1186/s12909-023-04388-w (PMC10262926; doi:10.1186/s12909-023-04388-w)
Supplement: Supplementary file 1 — Supplementary Material 1 [file 12909_2023_4388_MOESM1_ESM.docx]

Appendix 1:

The final questionnaires result of group a: Faculty members (n=11); group b: Residents (n=42); group c: Interns/Students (n=54).

| **NO** | **Questions** | **Strongly Disagree** | **Disagree** | **Neutral** | **Agree** | **Strongly Agree** |
| --- | --- | --- | --- | --- | --- | --- |
| **Group a: Faculty member** | | | | | | |
| 1 | Satisfaction with the platform and facilities for using e-classes | 0% | 9.09% | 9.09% | 63.63% | 18.18% |
| 2 | Increasing the quality of education (knowledge) for Medical Students | 0% | 18.18% | 18.18% | 54.54% | 9.09% |
| 3 | Increasing the quality of education (knowledge) for Medical Interns | 0% | 9.09% | 63.63% | 9.09% | 18.18% |
| 4 | Increasing the quality of education (knowledge) for Orthopedic Residents | 0% | 27.27% | 9.09% | 36.36% | 27.27% |
| 5 | Improving the quality of education (skills) for Medical Students | 9.09% | 36.36% | 27.27% | 9.09% | 9.09% |
| 6 | Improving the quality of education (skills) for Medical Interns | 0% | 36.36% | 45.45% | 9.09% | 9.09% |
| 7 | Improving the quality of education (skills) for Orthopedic Residents | 27.27% | 27.27% | 9.09% | 36.36% | 0% |
| 8 | Increasing discussion opportunities | 0% | 27.27% | 0% | 36.36% | 36.36% |
| 9 | Increasing the quality of training and participation in morning sessions | 0% | 27.27% | 9.09% | 27.27% | 36.36% |
| 10 | Improving educational curriculum | 9.09% | 18.18% | 36.36% | 27.27% | 9.09% |
| 11 | Improving evaluations and assignments | 9.09% | 36.36% | 9.09% | 27.27% | 18.18% |
| 12 | Increasing the quality of training in virtual shifts | 0% | 27.27% | 27.27% | 45.45% | 0% |
| 13 | Increasing the quality of education by flexible curriculum of shifts | 0% | 18.18% | 36.36% | 36.36% | 9.09% |
| 14 | Increasing the quality of journal club meetings | 9.09% | 18.18% | 18.18% | 27.27% | 27.27% |
| 15 | Increasing participation in research | 0% | 9.09% | 18.18% | 27.27% | 45.45% |
| 16 | Improving time and cost savings | 0% | 0% | 9.09% | 45.45% | 45.45% |
| 17 | Satisfaction with educational management | 9.09% | 9.09% | 18.18% | 27.27% | 36.36% |
| 18 | Satisfaction with research management | 9.09% | 0% | 18.18% | 36.36% | 36.36% |
| 19 | Satisfaction with the educational situation | 0% | 18.18% | 36.36% | 27.27% | 18.18% |
| 20 | Satisfaction with the treatment status | 0% | 18.18% | 54.54% | 27.27% | 0% |
| 21 | Using e-learning courses along with the post-crisis face-to-face courses after the crisis | 0% | 0% | 18.18% | 18.18% | 63.63% |
| 22 | Using e-learning courses alone after the crisis | 45.45% | 36.36% | 9.09% | 9.09% | 0% |
| **Group c: Interns/Students** | | | | | | |
| 1 | Satisfaction with the platform and facilities for using e-classes | 0% | 2.38% | 2.38% | 52.38 | 42.85% |
| 2 | Increasing the quality of education (knowledge) | 2.38% | 11.90% | 4.76% | 42.85% | 38.09% |
| 3 | Improving the quality of education (skills) | 7.14% | 21.42% | 26.19% | 26.19% | 19.04% |
| 4 | Increasing discussion opportunities | 2.38% | 19.04% | 11.90% | 28.57% | 38.09% |
| 5 | Increasing the opportunity to reflect and re-examine educational contents | 0% | 16.66% | 9.52% | 33.33% | 40.47% |
| 6 | Increasing the quality of training and participation in morning sessions | 2.38% | 26.19% | 4.76% | 28.57% | 38.09% |
| 7 | Improving educational curriculum | 2.38% | 14.28% | 16.66% | 23.80% | 42.85% |
| 8 | Increasing learning of orthopedic contents (due to reduced presence in the ward) | 2.38% | 14.28% | 16.66% | 19.04% | 47.61% |
| 9 | Increasing the rate of orthopedic study | 4.76% | 4.76% | 9.52% | 26.19% | 54.76% |
| 10 | Improving evaluations and assignments | 2.38% | 2.38% | 21.42% | 33.33% | 40.47% |
| 11 | Increasing the quality of training in virtual shifts | 0% | 11.90% | 33.33% | 26.19% | 28.57% |
| 12 | Increasing the quality of education by flexible shifts curriculum | 2.38% | 7.14% | 19.04% | 28.57% | 42.85% |
| 13 | Increasing the quality of journal club meetings | 2.38% | 9.52% | 11.90% | 21.42% | 54.76% |
| 14 | Increasing participation in research | 0% | 2.38% | 19.04% | 26.19% | 52.38% |
| 15 | Increasing the possibility of communication with faculty members | 0% | 14.28% | 23.80% | 21.42% | 40.47% |
| 16 | Improving time and cost savings | 0% | 2.38% | 9.52% | 33.33% | 54.76% |
| 17 | Reducing stress levels and mental conflicts | 0% | 2.38% | 9.52% | 28.57% | 59.52% |
| 18 | Satisfaction with educational management | 2.38% | 4.76% | 19.04% | 28.57% | 45.23% |
| 19 | Satisfaction with the educational situation | 4.76% | 9.52% | 11.90% | 35.71% | 38.09% |
| 20 | Satisfaction with the treatment status | 2.38% | 19.04% | 35.71% | 14.28% | 28.57% |
| 21 | Using e-learning courses along with the post-crisis face-to-face courses after the crisis | 0% | 2.38% | 14.28% | 26.19% | 57.14% |
| 22 | Using e-learning courses alone after the crisis | 11.90% | 33.33% | 4.76% | 16.66% | 33.33% |
| **Group b: Residents** | | | | | | |
| 1 | Satisfaction with the platform and facilities for using e-classes | 5.55% | 5.55% | 1.85% | 57.40% | 29.62% |
| 2 | Increasing the quality of education (knowledge) for Students | 11.62% | 16.27% | 16.27% | 34.88% | 20.93% |
| 3 | Increasing the quality of education (knowledge) for Interns | 9.09% | 9.09% | 36.36% | 54.54% | 0% |
| 4 | Improving the quality of education (skills) for Students | 37.20% | 13.95% | 34.88% | 6.97% | 6.97% |
| 5 | Improving the quality of education (skills) for interns | 9.09% | 9.09% | 27.27% | 54.54% | 0% |
| 6 | Increasing discussion opportunities | 14.81% | 18.51% | 11.11% | 37.03% | 18.51% |
| 7 | Increasing the opportunity to reflect and re-examine educational contents | 11.11% | 7.40% | 3.70% | 46.29% | 31.48% |
| 8 | Increasing the quality of training and participation in morning sessions | 9.25% | 14.81% | 42.59% | 24.07% | 9.25% |
| 9 | Increasing the rate of orthopedic study | 14.81% | 9.25% | 16.66% | 31.48% | 27.77% |
| 10 | Improving evaluations and assignments | 11.11% | 9.25% | 29.62% | 37.03% | 12.96% |
| 11 | Increasing participation in research | 14.81% | 12.96% | 37.03% | 20.37% | 14.81% |
| 12 | Improving time and cost savings | 5.55% | 5.55% | 7.40% | 46.29% | 35.18% |
| 13 | Reducing stress levels and mental conflicts | 3.70% | 7.40% | 7.40% | 25.92% | 55.55% |
| 14 | Using e-learning courses alongside post-crisis face-to-face courses after the crisis | 5.55% | 11.11% | 7.40% | 31.48% | 44.44% |
| 15 | Using e-learning courses alone after the crisis | 37.03% | 25.92% | 14.81% | 16.66% | 5.55% |
