# Supplementary material for: Medical education during the COVID-19 pandemic: lessons for the orthopedic departments
Source: BMC Med Educ. 2023 Jun 13;23:436. doi: 10.1186/s12909-023-04388-w (PMC10262926; doi:10.1186/s12909-023-04388-w)
Supplement: Supplementary file 2 — Supplementary Material 2 [file 12909_2023_4388_MOESM2_ESM.docx]

Appendix 2:

Participants responses to survey questions by Likert scale. Group a: Faculty members (n=11); Group b: Residents (n=42); Group c: Interns/Students (n=54).
